# Identification of Novel Serological Autoantibodies in Takayasu Arteritis Patients Using HuProt Arrays

**DOI:** 10.1074/mcp.RA120.002119

**Published:** 2021-02-03

**Authors:** Xiaoting Wen, Guang Song, Chaojun Hu, Jianbo Pan, Ziyan Wu, Liubing Li, Chenxi Liu, Xinping Tian, Fengchun Zhang, Jiang Qian, Heng Zhu, Yongzhe Li

**Affiliations:** 1 Department of Clinical Laboratory, Peking Union Medical College Hospital, Chinese Academy of Medical Sciences & Peking Union Medical College, Beijing, China; 2 Department of Rheumatology, Shanxi Bethune Hospital, Shanxi Academy of Medical Sciences, Taiyuan, Shanxi, China; 3 Department of Pharmacology and Molecular Sciences, Johns Hopkins University School of Medicine, Baltimore, Maryland, USA; 4 Department of Rheumatology and Clinical Immunology, Peking Union Medical College Hospital, Chinese Academy of Medical Sciences & Peking Union Medical College, Key Laboratory of Rheumatology and Clinical Immunology, Ministry of Education, Beijing, China; 5 Department of Ophthalmology, Wilmer Institute, Johns Hopkins University School of Medicine, Baltimore, Maryland, USA; 6 State Key Laboratory of Complex Severe and Rare Diseases, Peking Union Medical College Hospital, Chinese Academy of Medical Science & Peking Union Medical College, Beijing, China

**Keywords:** Takayasu arteritis, autoantibody, biomarker, protein microarray, TAK, Takayasu arteritis, ESR, erythrocyte sedimentation rate, CRP, C-reactive proteins, AAV, ANCA-associated vasculitis, SS, Sjögren's syndrome, RA, rheumatoid arthritis, SLE, systemiclupusery thematosus

## Abstract

To identify novel autoantibodies of Takayasu arteritis (TAK) using HuProt array-based approach, a two-phase approach was adopted. In Phase I, serum samples collected from 40 TAK patients, 15 autoimmune disease patients, and 20 healthy subjects were screened to identify TAK-specific autoantibodies using human protein (HuProt) arrays. In phase II, the identified candidate autoantibodies were validated with TAK-focused arrays using an additional cohort comprised of 109 TAK patients, 110 autoimmune disease patients, and 96 healthy subjects. Subsequently, the TAK-specific autoantibodies validated in phase II were further confirmed using western blot analysis. We identified and validated eight autoantibodies as potential TAK-specific diagnostic biomarkers, including anti-SPATA7, -QDPR, -SLC25A2, -PRH2, -DIXDC1, -IL17RB, -ZFAND4, and -NOLC1 antibodies, with AUC of 0.803, 0.801, 0.780, 0.696, 0.695, 0.678, 0.635, and 0.613, respectively. SPATA7 could distinguish TAK from healthy and disease controls with 73.4% sensitivity at 85.4% specificity, while QDPR showed 71.6% sensitivity at 86.4% specificity. SLC25A22 showed the highest sensitivity of 80.7%, but at lower specificity of 67.0%. In addition, PRH2, IL17RB, and NOLC1 showed good specificities of 88.3%, 85.9%, and 86.9%, respectively, but at lower sensitivities (<50%). Finally, DIXDC1 and ZFAND4 showed moderate performance as compared with the other autoantibodies. Using a decision tree model, we could reach a specificity of 94.2% with AUC of 0.843, a significantly improved performance as compared with that by each individual biomarker. The performances of three autoantibodies, namely anti-SPATA7, -QDPR, and -PRH2, were successfully confirmed with western blot analysis. Using this two-phase strategy, we identified and validated eight novel autoantibodies as TAK–specific biomarker candidates, three of which could be readily adopted in a clinical setting.

## Highlights

• HuProt array and TAK-focused arrays were adopted to identify TAK-specific biomarkers.
• Eight autoantibodies were identified as potential TAK-specific biomarkers.
• The decision tree model could significantly improve biomarker performance.
• Anti-SPATA7, -QDPR, and -PRH2 might be potentially adopted in a clinical setting.

## Graphical Abstract

**Figure:**
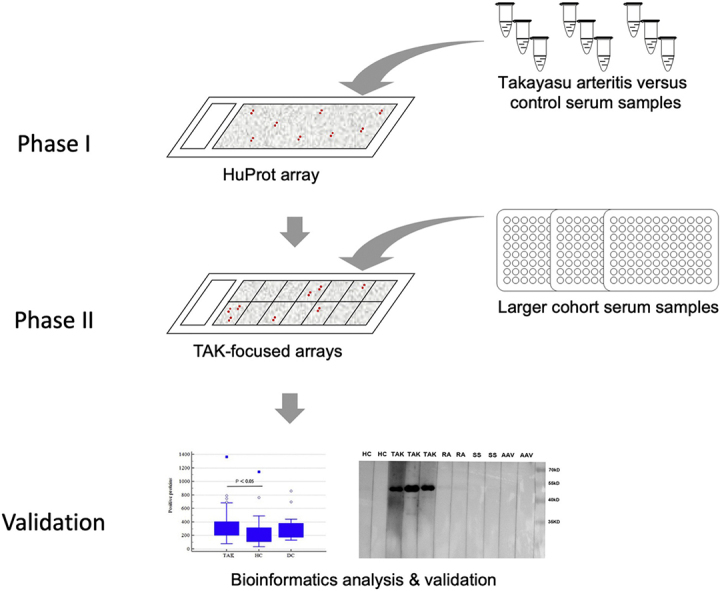

## In Brief

A two-phase approach was adopted to identify novel autoantibody biomarkers of Takayasu arteritis (TAK) patients. HuProt array together with a TAK-focused array identified eight novel autoantibodies as TAK-specific biomarker candidates. Of them, the anti-SPATA7, -QDPR, and -PRH2 have the potential to be readily adopted in a clinical setting.

Takayasu arteritis (TAK) is an autoimmune disease characterized by chronic vasculitis, which mainly affects the aorta and its main branches, especially the brachiocephalic, carotid, and subclavian arteries. Although TAK is known as a rare disease with a disproportionately high incidence in East Asia, it has been recently observed in all ethnicities worldwide (1). Because it typically shows nonspecific symptoms, significant delay in TAK diagnosis is common (2, 3).

To facilitate early diagnosis, imaging methods, such as computerized tomography and magnetic resonance angiography, have been developed. In addition, erythrocyte sedimentation rate (ESR) and C-reactive proteins (CRP) that are acute-phase response reactants were used to monitor TAK activity in clinic, although they cannot reliably quatify disease activity (4). Serum biomarkers, such as matrix metalloproteinase-9, TNF-α, and IL-6 (5, 6), were found to be increased in TAK, as well as other inflammatory diseases. Moreover, serum antibodies, such as anti-endothelial, -aorta, -ferritin, -annexin V, and -monocyte antibodies, have been reported (7, 8, 9, 10, 11), although they were not highly specific in TAK diagnosis.

Although pentraxin 3, which is known to be produced at inflammation sites in response to proinflammatory signals, was identified as a biomarker for TAK and its progression (12, 13), it is still unclear whether it plays a role in TAK patients exhibiting either an active or a dormant disease stage (14). Autoantibody tests have significantly contributed toward the diagnosis of many autoimmune diseases (15, 16, 17). In addition, they have been reported to be used in disease prediction, prognosis, and prevention (18). To date, however, no definitive autoantibody for use in TAK clinical diagnosis has been reported.

Recent advances in protein microarray technology, especially HuProt arrays, have enabled unbiased screening of autoantibodies at the human proteome level. In the past, we have successfully used this method to identify and validate autoantibodies as biomarkers for several autoimmune diseases, such as primary biliary cirrhosis and Behcet's disease (15, 17). Similarly, in this study we employed the two-phase strategy involving HuProt and TAK-focused arrays in phase I and phase II, respectively, to identify and validate novel TAK biomarkers.

## Experimental Procedures

### Patients and Study Design

**Fig. 1:**
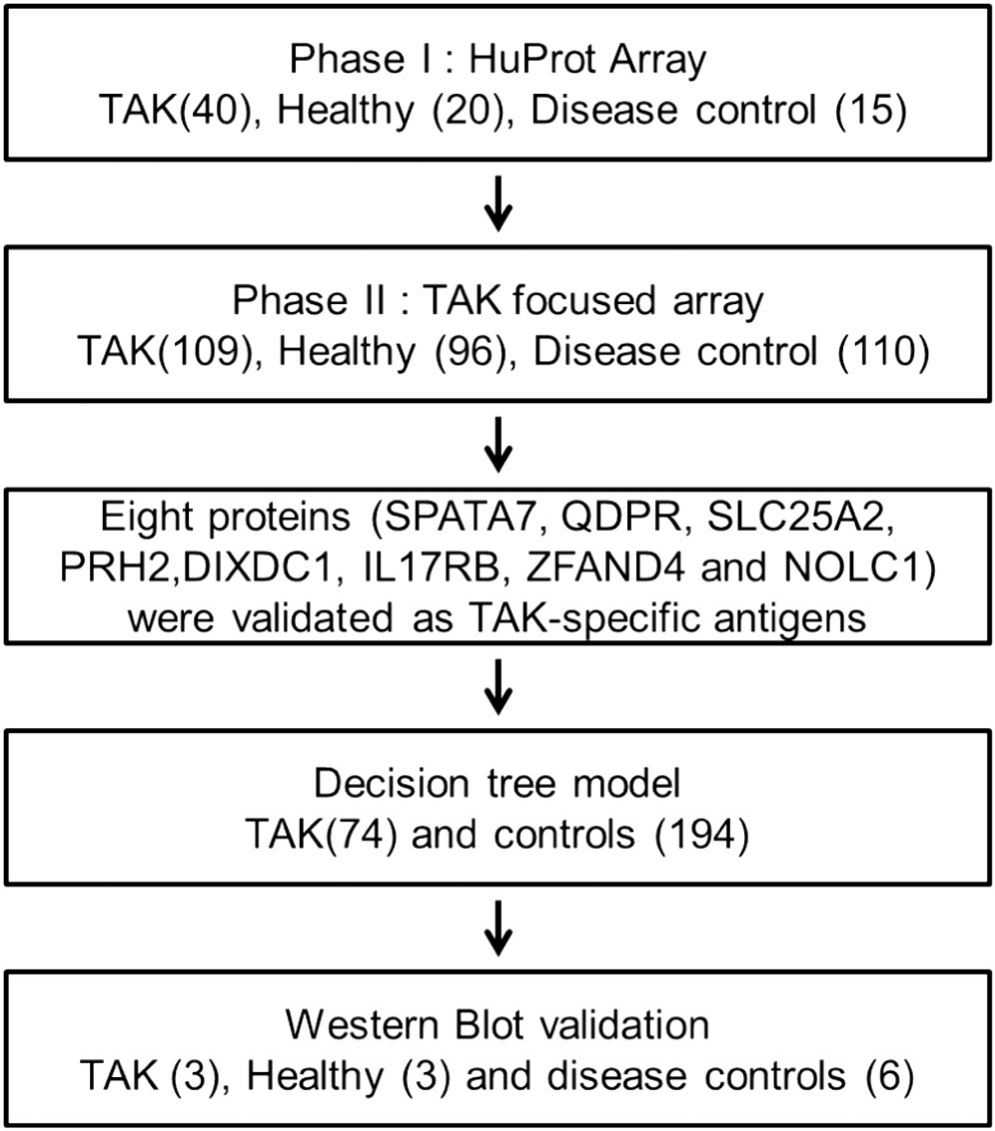
**Flow chart of the overall study design**.

To identify novel biomarkers more efficiently with the HuProt array system, a two-phase strategy, comprised of discovery (Phase I) and verification (Phase II) phases, with a small modification (*i.e.*, western blot validation phase) was used in this study (Fig. 1). In phase I, TAK-associated autoantigens were unbiasedly screened with HuProt arrays in a small sample size including 40 TAK and 15 autoimmune disease patients (five Sjögren's syndrome, five ANCA - associated vasculitis, and five systemic lupus erythematosus), and 20 healthy subjects. In phase II, the candidates identified in phase I were validated in a larger cohort, comprised of 109 TAK patients, 110 autoimmune disease controls, and 96 healthy controls. In total, 149 TAK-positive patients were identified from 390 patient serum samples according to TAK diagnosis and classification criteria of the American College of Rheumatology in 1990 (19). They were then divided into active (*n* = 101) and stable (*n* = 48) groups according to Kerr's criteria (20) (Table 1). Both TAK patients and controls were from Peking Union Medical College Hospital between February 2012 and June 2014. Serum samples were collected from the cubital vein under fasting conditions in the morning. Blood samples were drawn and dispensed into 5-mL procoagulation tubes with gel, then centrifuged at 1000*g* for 5 min. Serum samples were aliquoted and frozen at –80 °C, and repeated freezing and thawing were avoided before use. This study was approved by the Ethics Committee of Peking Union Medical College Hospital.

**Table 1:** Clinical characteristics of TAK patients

Characteristic	Phase I	Phase II
TAK patients/controls	40/35	109/206
Age (y)	30.9 ± 10.4	31.1 ± 10.9
Female/male	32/8	14/95
ESR (mm/h)	14 (7–34)	11 (5–26)
hsCRP (mg/L)	4.06 (1.10–13.14)	3.44 (0.58–22.48)
Stable/active patients	23/17	78/31

ESR, erythrocyte sedimentation rate; hsCRP, high-sensitivity C-reactive protein; TAK, Takayasu arteritis.

ESR and hsCRP were showed as median (lower quartile-upper quartile).

### Construction and Quality Control of Protein Arrays

HuProt and TAK-focused arrays were employed in phase I and II studies, respectively. Each HuProt array (CDI Laboratories) is comprised of 20,240 individual purified full-length human proteins as previously described (15). Each TAK-focused array (CDI Laboratories) included 43 purified proteins that were identified as potential biomarkers in phase I. The proteins used for both arrays were GST-tagged at their N termini and printed in duplicate on a glass substrate (OPEpoxySlide). Thus, the quality of the protein arrays can be verified on the basis of the fluorescence intensity of the anti-GST signals, detected with anti-GST antibodies and subsequent Cy5-labeled secondary antibody staining. The number of detected proteins and consistency of duplicate protein signals were adapted to control the microarray quality.

### Serum Profiling of HuProt Arrays

In phase I, each HuProt array was blocked with 3% w/v BSA (Sigma) in PBST buffer for 1.5 h. Next, 180 μl of 1000-fold diluted serum sample (in PBST buffer with 3% w/v BSA) was added and incubated for 1 h with gentle shaking. The arrays were then washed in PBST, followed by incubation with 180 μl of goat anti-human IgG conjugated to Alexa 647 (1:1000 fold dilution; The Jackson Laboratory) for 1 h with gentle shaking. After rinsing with PBST buffer followed by deionized water, the slides were dried and scanned with the GenePix 4000B Microarray Scanner (Molecular Devices).

### Serum Profiling of TAK-Focused Arrays

In phase II, the assay procedure was similar to that as described for the HuProt microarray, except that a single slide was divided into 12 identical blocks by a 12-hole rubber gasket so that 12 samples on each chip can be tested simultaneously. Fifty microliters of diluted serum (1:1000) and detection antibodies were added to each block and incubated for 1 h with gentle shaking.

### Western Blot Analysis

Purified proteins, PRH2 (proline rich protein Hae III subfamily 2; 44 kDa), QDPR (dihydropteridinereductase; 53 kDa), and SPATA7 (spermatogenesis associated 7; 95 kDa) (provided by CDI), were separated by SDS-PAGE (EZBiolab) followed by blotting onto polyvinylidene fluoride membranes. The membranes were first blocked with 5% BSA in PBST buffer and then incubated with serum (1:200 fold dilution in PBST buffer with 5% BSA) or mouse anti-GST antibody as positive control for 2 h. Subsequently, the membranes were washed with TBST buffer three times and were incubated with HRP-conjugated goat anti-human IgG (EASYBIO; 1:5000) or anti-mouse IgG for 1 h. Enhanced chemiluminescence substrate was used for detection, and the blots were visualized using Clinx Chemic Capture (Clinx Science Instruments).

### Data Processing and Statistical Analysis

The image processing and data acquisition of both arrays were performed using GenePix Pro 6.0 (Molecular Devices). The mean of the median foreground to median background signal ratio was calculated for each protein. Proteins in the HuProt arrays that satisfied the following criteria were considered target TAK candidate proteins: (1) a cutoff value of peak + 8 standard deviations (SD) for each protein in healthy controls; and (2) those with *p* ≤ 0.05 between different groups (TAK/control, TAK/healthy, and TAK/disease control) after applying the significant analysis of microarray (SAM) algorithm on the Gene Pattern platform (21). For the TAK-focused arrays, the maximum and minimum fold values of each protein were divided into 1000 points, and the cutoff value of each protein was defined as the point with the highest sensitivity and specificity. Thus, proteins with an ideal area under the curve (AUC) of 0.60 were considered as TAK-specific autoantigens. The positive rate of HuProt arrays among groups (TAK patients, autoimmune disease controls, and healthy controls) and that between active and stable TAK groups were evaluated using a Chi-squared test. In addition, the differences of ESR and hsCRP in active and stable TAK groups were subjected to Mann–Whitney test. To predict the diagnostic value of antigens screened with TAK-specific arrays, decision tree analysis using the C4.5 algorithm was performed. A value of *p* < 0.05 is considered statistically significant.

## Results

### Characteristics of Subjects

A total of 149 TAK patients (31.1 ± 10.7 years old; 85.2% [127/149] females) were recruited in this study. They were divided into active (*n* = 101) and stable (*n* = 48) groups according to Kerr's criteria (Table 1). In total, 241 non-TAK subjects were grouped into the healthy control group (*n* = 116; 37.6 ± 8.9 years old; 33.6% females) and the autoimmune disease control group, which was comprised of AAV (ANCA-associated vasculitis; *n* = 37; 55.3 ± 16.4 years old; 51.4% females), SS (Sjögren's syndrome; *n* = 28; 50.8 ± 15.2 years old; 67.9% females), RA (rheumatoid arthritis; *n* = 55; 49.2 ± 13.0 years old; 65.5% females), and SLE (systemiclupusery thematosus; *n* = 5; 40.6 ± 7.2 years old; 100% females).

### Quality Assessment of Protein Arrays

To assess the quality of the protein arrays before performing the serum profiling assays, randomly selected HuProt and TAK-focused arrays were probed with anti-GST antibodies, and analyses of the obtained signal intensity values revealed a 93.2% detection rate with a correlation coefficient of 0.978 between the duplicate spots of the same protein for the HuProt arrays (Y = 0.99X + 0.53; R^2^ = 0.96), and a 100% detection rate with a correlation coefficient of 0.880 for the TAK-focused arrays (Y = 0.91X + 2.74; R^2^ = 0.77), suggesting reliable reproducibility (supplemental Fig. 1). Thus, both arrays passed the quality control tests.

### Screening for TAK-Associated Autoantigens With HuProt Arrays

**Fig. 2:**
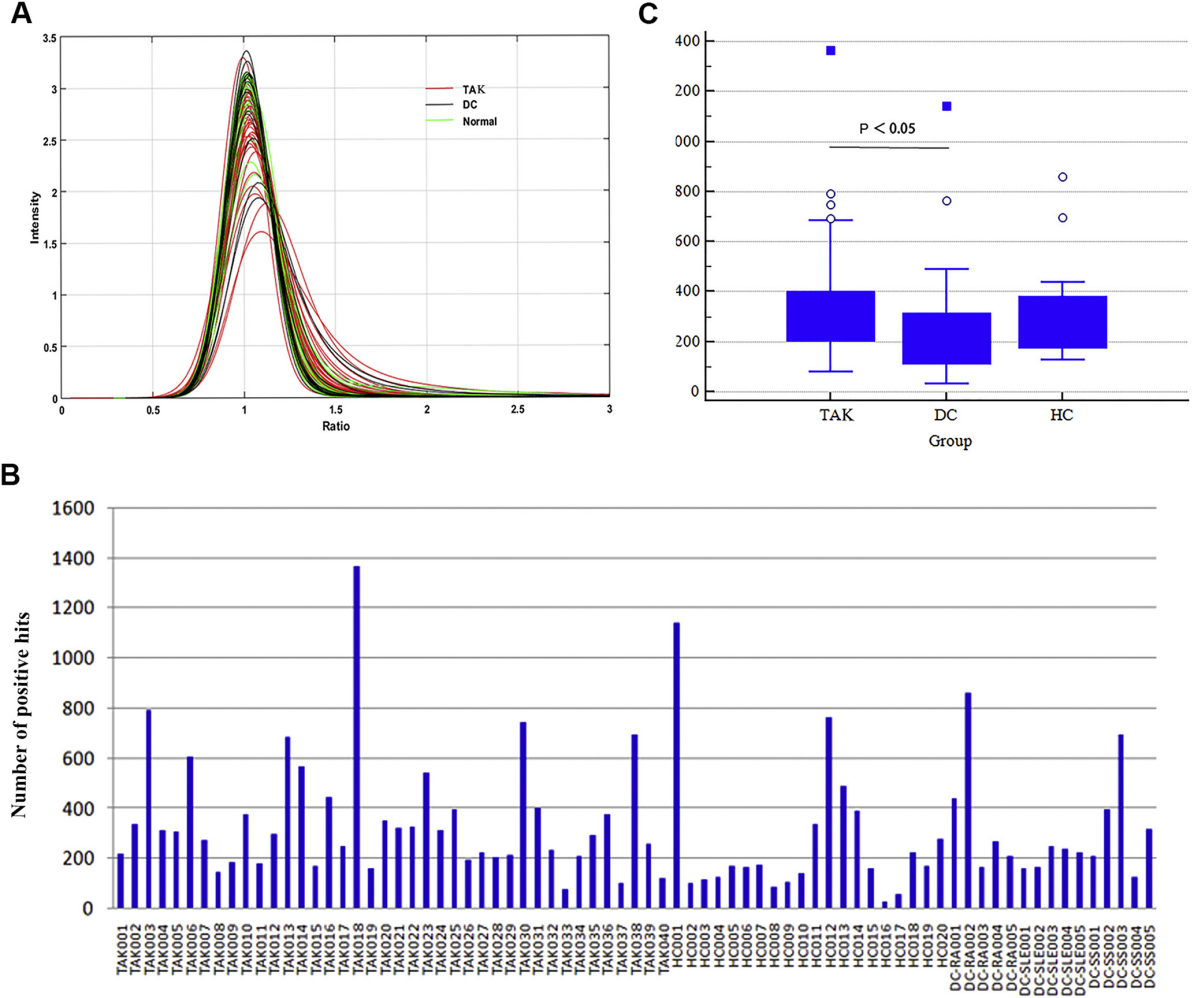
**Global analysis of the serum profiling assays on HuProt.***A*, histogram analysis of signal intensity of all the proteins on HuProt arrays in the serum profiling assays (75 serum samples). Using a cutoff value of 8 SD determined using the control sera, autoantigens recognized by IgG autoantibodies (positives) were identified. *B*, distribution of positives identified in each of the 75 serum samples, ranging from <100 to >1000. *C*, boxplot analysis of positives identified in the TAK, healthy, and disease control groups. The median of positive autoantigens (303) identified in the TAK group is significantly higher than that obtained in the healthy group (168). No significant differences were observed between the TAK and disease control groups or between the healthy and disease control groups. AAV, ANCA-associated vasculitis; DC, disease control; HC, healthy control; RA, rheumatoid arthritis; SLE, systemic lupus erythematosus; SS, primary Sjögren's syndrome; TAK, Takayasu arteritis.

In phase I of the two-phase strategy, HuProt arrays were individually incubated with serum samples collected from 40 TAK patients, 15 autoimmune disease (*i.e.*, five RA, five SLE, five SS) patients, and 20 healthy subjects to identify TAK-associated autoantigens (Fig. 1). The signal intensity of the majority of the human proteins on the HuProt arrays was around 1 after normalization across all of the 75 sera samples, indicating that these human proteins were not immunogenic (Fig. 2*A*). Using a stringent cutoff value of 8 standard deviations, IgG isotype-positive proteins were identified as candidate autoantigens for each serum sample. Statistical analysis revealed that the number of identified positives varied dramatically, ranging from <100 to >1000 proteins per serum sample (Fig. 2*B*). Using boxplot analysis, the median of the TAK group was found to be 303, which was significantly higher than that (168) of the healthy control group. No significant difference was found between the TAK and disease control groups (240) or between the healthy and disease control groups (*P* > 0.05) (Fig. 2*C*).

**Fig. 3:**
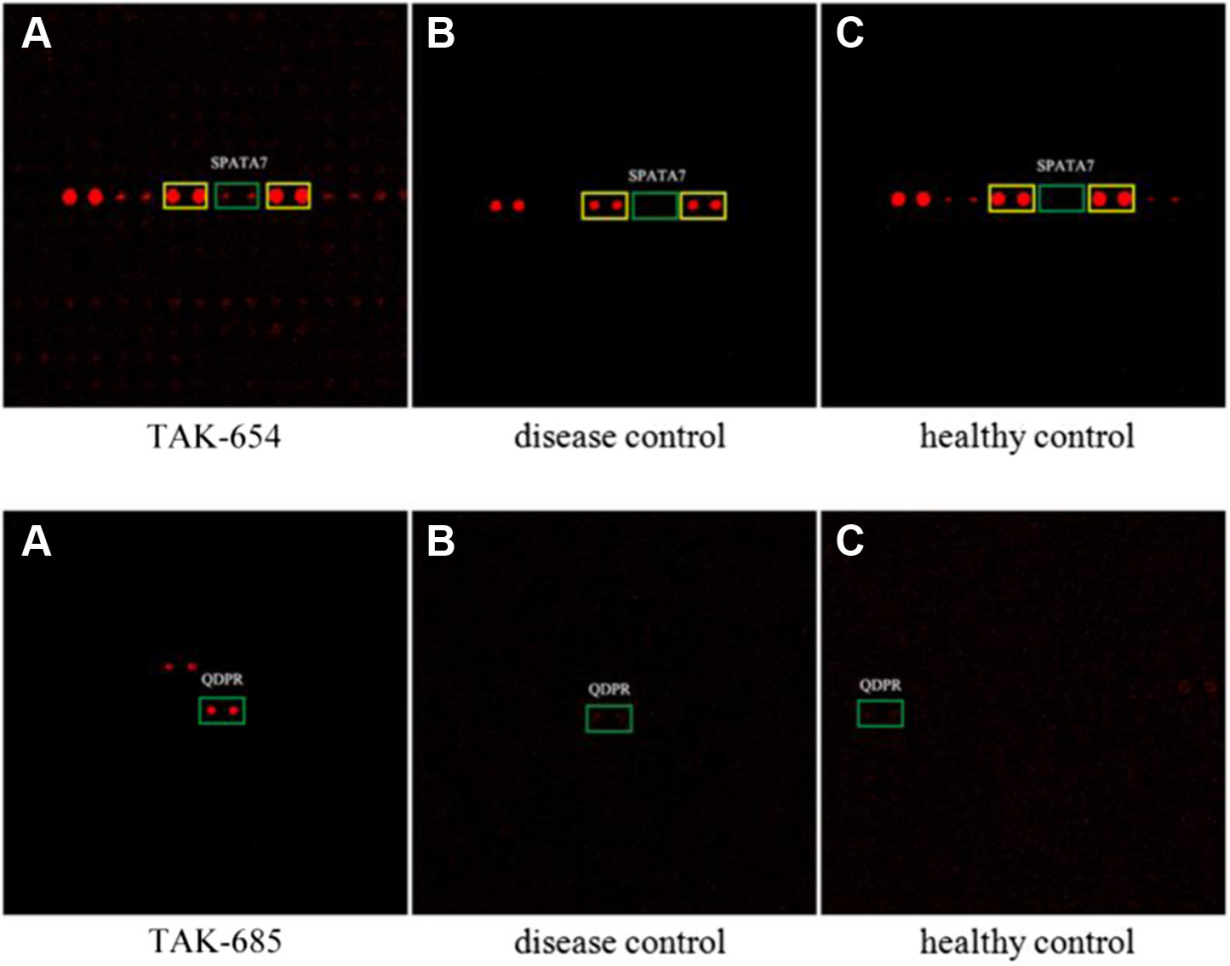
**Representative images of candidate autoantigens identified in HuProt arrays in phase I.** Anti-SPATA7 and -QDPR autoantibodies were observed in the TAK sample (*A*) but not in the disease control (*B*) or healthy control samples (*C*). *Green boxes* indicate candidate autoantigens; yellow boxes indicate positive control proteins. TAK, Takayasu arteritis.

Using SAM analysis, 43 autoantigens were identified as TAK-associated autoantigens with *P* ≤ 0.05 (supplemental Table S1). Among them, HuProt array images of SPATA7 and QDPR were shown as representative examples in Figure 3. Except ESR2, the majority of these proteins have not been previously reported to associate with any autoimmune diseases. Overall, these newly identified candidate proteins were annotated for diverse biological functions, and Gene Ontology (GO) analysis did not reveal any enriched GO terms (22). Nonetheless, some of the 43 candidate autoantigens already showed good sensitivity at >80% specificity in phase I. For example, anti-QDPR autoantibodies were found in all of the 40 TAK serum samples tested, while anti-DIXDC1 autoantibodies showed 90% sensitivity, suggesting that some of them might serve as promising TAK biomarkers.

### Validation of TAK-Associated Autoantigens With TAK-Focused Arrays

To ensure reproducibility and to avoid a potential overfitting problem, the 43 candidates identified in phase I were repurified and spotted in a 2 × 6 subarray format to form a TAK-focused array for phase II validation with a larger cohort of 315 serum samples. The data was normalized and the AUC values for each candidate protein were calculated (supplemental Table S2). Eight proteins, SPATA7, QDPR, SLC25A22 (solute carrier family 25 member 22), PRH2, DIXDC1 (DIX domain containing 1), IL17RB (interleukin 17 receptor B), ZFAND4 (finger AN1-type containing zinc 4), and NOLC1 (nucleolar and coiled-body phosphoprotein 1), which showed AUC of 0.803, 0.801, 0.780, 0.696, 0.695, 0.678, 0.635, and 0.613, respectively, were validated as TAK-specific autoantigens (Table 2 and supplemental Fig. S2). SPATA7 and QDPR showed the highest AUC (>0.8) among the validated autoantigens. SPATA7 could distinguish TAK from healthy and disease control groups with 73.4% sensitivity at 85.4% specificity; whereas QDPR showed 71.6% sensitivity at 86.4% specificity. Of note, SLC25A22 showed the highest sensitivity of 80.7% but at a lower specificity of 67.0%. On the other hand, PRH2, IL17RB, and NOLC1 showed good specificities (>85%) but at lower sensitivities (<50%). Boxplot analysis of the top four autoantigens demonstrated that the median values of the signal intensity obtained in the TAK group were significantly higher than those obtained in the negative control group (supplemental Fig. S3). It is noteworthy that the performance of these eight proteins in phase II validation was not completely consistent with that observed in phase I, suggesting the necessity to validate candidate biomarkers with a larger cohort (*e.g.*, >200).

**Table 2:** Validated TAK biomarkers with the highest AUC values

Candidates	AUC	Accuracy (%)	Sensitivity (%)	Specificity (%)
SPATA7	0.803	81.3	73.4	85.4
QDPR	0.801	81.3	71.6	86.4
SLC25A22	0.780	71.8	80.7	67.0
PRH2	0.696	74.0	46.8	88.3
DIXDC1	0.695	66.0	67.0	65.5
IL17RB	0.678	72.1	45.9	85.9
ZFAND4	0.635	68.3	50.5	77.7
NOLC1	0.613	69.2	35.8	86.9

### A Decision Tree Model Constructed for TAK Diagnosis

**Fig. 4:**
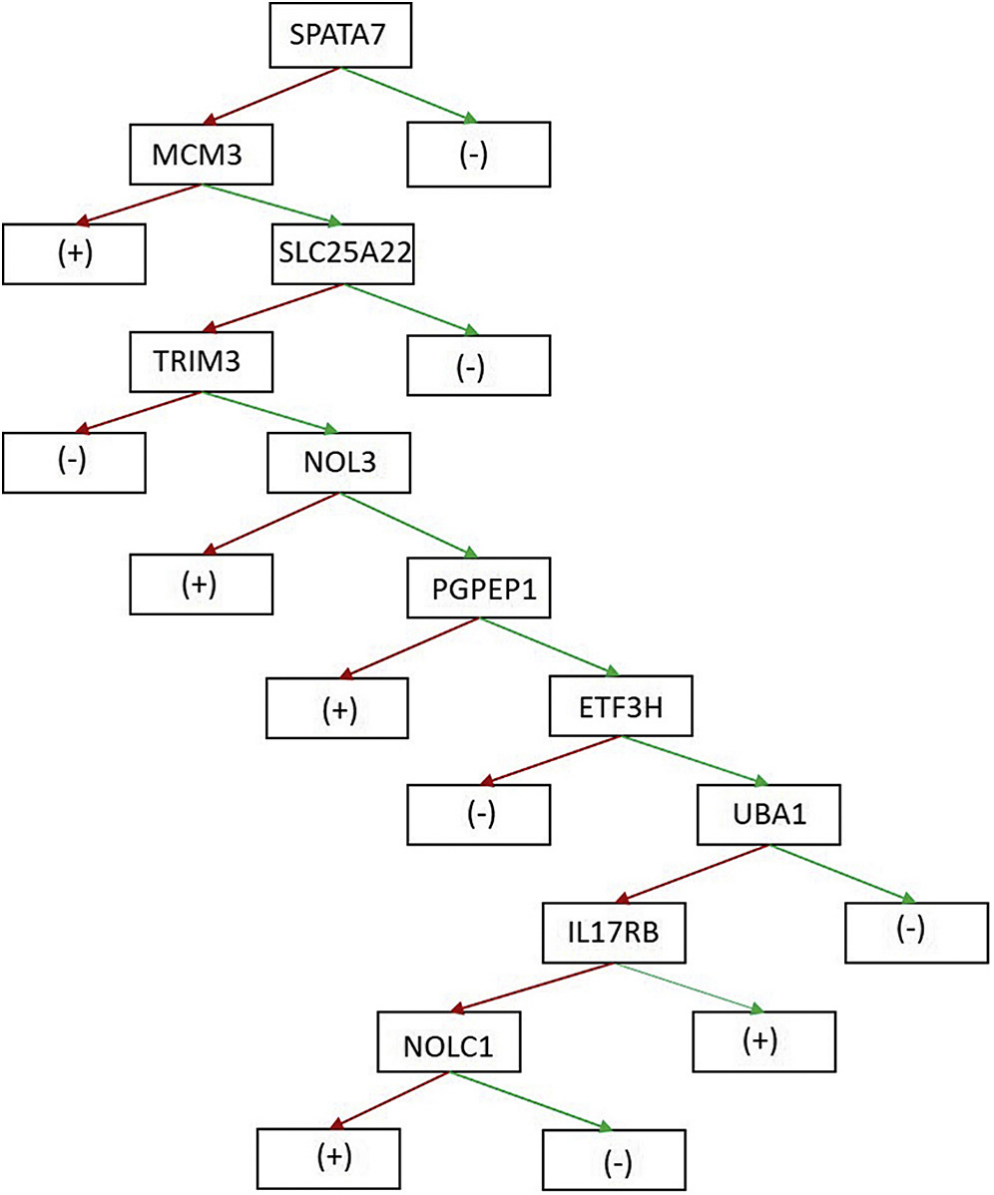
**TAK diagnosis decision tree constructed based on antigens validated in phase II.***Red arrow* antigen with signal value ≥cutoff; *green arrow* antigen with signal value ≤cutoff; (+): TAK cases; (–): controls.

An optimal decision tree model is comprised of ten antigens capable of detecting 74 TAK cases and 194 controls (Fig. 4). The constructed decision tree model showed a sensitivity of 68.9% at a specificity of 94.2% with an AUC value of 0.843. The specificity and AUC value of the decision tree model were significantly higher compared with those of single markers with the highest values (86.9% specificity of NOLC1, 0.803 AUC of SPATA7), while maintaining its sensitivity higher than the majority of the validated biomarkers, such as PRH2, DIXDC1, IL17RB, ZFAND4, and NOLC1.

The age of identified 74 TAK patients was 31.3 ± 10.4 years old, and the ratio of male/female and active/stable TAK patients was 0.18 (11/63) and 0.40 (21/53), respectively. The median of ESR was 10 mm/h (lower quartile-upper quartile: 4–23 mm/h), while the median of hsCRP was 2.1 mg/L (lower quartile-upper quartile: 4–23 mg/L). These characters were not significantly different from 109 TAK patients in phase II. Twelve controls showed positive results including six AAV and six healthy controls indicating there might be similar pathogenesis between the vasculitis of TAK and AAV.

### Association Between Serum Autoantibodies and TAK Activity

The expression levels of both ESR and CRP, which are commonly used to monitor disease conditions clinically, were significantly elevated in the active TAK group compared with the stable group (27 mm/h vs 9 mm/h; 18 mg/l vs 2.17 mg/l). However, none of the eight validated autoantigens showed any significant difference in TAK diagnostic potential between the TAK active and stable groups (Table 3), neither did the remaining candidates (supplemental Table S1)

**Table 3:** Association between eight biomarkers and TAK activity

Protein	TAK stable (78)		TAK active (31)		χ^2^	*p*
	Positive number	Positive rate (%)	Positive number	Positive rate (%)		
SPATA7	58	74.4	22	71.0	0.13	0.72
QDPR	58	74.4	20	64.5	1.06	0.30
SLC25A22	64	82.1	24	77.4	0.31	0.58
PRH2	33	42.3	18	58.1	2.21	0.14
DIDXDC1	53	67.9	20	64.5	0.12	0.73
IL17RB	36	46.2	14	45.2	0.009	0.96
ZFAND4	38	48.7	17	54.8	0.33	0.56
NOLC1	26	33.3	13	41.9	0.71	0.40

### Biomarker Candidate Validation by Western Blot Analysis

**Fig. 5:**
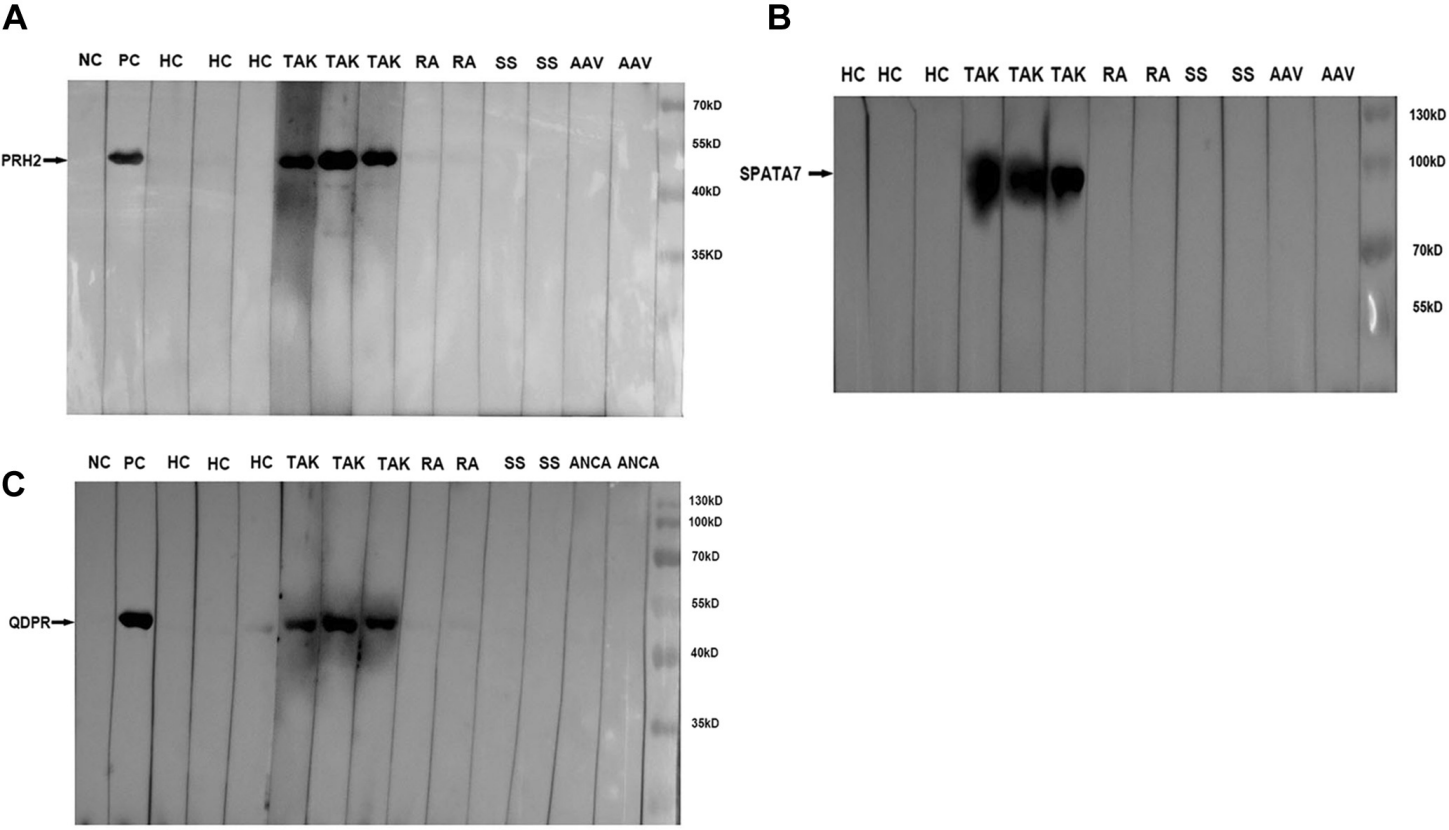
**Western blot validation of PRH2, SPATA7, and QDPR autoantibodies.** Western blot analysis confirmed the presence of autoantibodies against (*A*) PRH2 (44 kD), (*B*) SPATA7 (95 kD), and (*C*) QDPR (53kD) in three TAK serum samples, but not in the healthy or disease control samples. Autoantibody tests against PRH2, SPATA7, and QDPR were performed simultaneously and shared positive controls. AAV, ANCA-associated vasculitis; HC, healthy control; NC, negative control; PC, positive control of anti-GST antibody; RA, rheumatoid arthritis; SS, primary Sjögren's syndrome; TAK, Takayasu arteritis.

Although protein array technology has shown great promise in biomarker discovery and validation, it has yet to be widely adopted in clinical settings. Hence, we aim to facilitate the utilization of microarray-based tests in clinical practice. Purification of four candidate proteins with the top AUC values in phase II was attempted. With the exception of SLC25A22, sufficient amounts of SPATA7, QDPR, and PRH2 proteins were obtained, and western blot analysis of these three proteins was subsequently performed. Each of these proteins was incubated with serum samples collected from three representative TAK patients and three healthy subjects, as well as six disease controls (two RA patients, two SS patients, and two AAV patients). The basic characters of these subjects were depicted in supplemental Table S2. Autoantibodies targeting PRH2, QDPR, and SPATA7 were readily detected in the three TAK patient samples, but not in the healthy or disease control samples (Fig. 5). Therefore, these three newly identified proteins hold a great potential to serve as biomarkers in a clinical setting.

## Discussion

We discovered and validated eight autoantigens as TAK-specific diagnostic biomarkers using a two-phase approach. Forty-three candidate autoantigens were identified in the phase I screening using the HuProt arrays, which have a >80% coverage of the human proteome. In phase II, eight proteins, namely SPATA7, QDPR, SLC25A2, PRH2, DIXDC1, IL17RB, ZFAND4, and NOLC1, were validated as TAK-specific serological biomarkers *via* screening the TAK-focused arrays using an additional large cohort of 390 serum samples. Furthermore, SPATA7, QDPR, and PRH2 were further confirmed by western blot to be TAK-specific autoantigens. Hence, these proteins can be immediately adopted for use in clinical applications.

The number of autoantigens identified in HuProt arrays varied more than tenfold from sample to sample in each group. This phenomenon suggested the heterogeneity of antigen-specific immune responses between individuals. However, the median of the identified positives with the TAK samples was significantly higher than that of healthy controls, suggesting that the occurrence of vascular injury in TAK patients might be a common cause of the elevated autoimmune responses. In contrast to SLE, RA, and SS patients, the primary sites affected in TAK patients were large vessels, where autoantigen secretion and autoantibody recognition are presumably easy.

To ensure reproducibility and avoid potential overfitting problems, a larger cohort was used in phase II validation. Only eight proteins were validated with good sensitivity (>70%) at decent specificity (>65%). The annotated functions of these autoantigens are diverse. IL-17RB is the receptor of IL-17B and IL-17E. The binding of IL-17RB and IL-17B can mediate NF-κB activation and lead to the production of proinflammatory cytokines (23). Elevated IL-17B levels have been reported in the synovial tissues of rheumatoid arthritis patients, which can promote TNF-α-induced G-CSF and IL-6 expression in fibroblasts (24). On the other hand, IL-17E/IL-17RB interaction can promote Th2 responses, regulate B cell function, and induce Th17-driven autoimmune inflammation (25). In SS patients, it was reported to participate in pathogenesis by promoting inflammation (26). Thus, we speculated that anti-IL-17RB antibodies may act as agonist of IL-17RB and play a potentially similar proinflammatory role in TAK patients.

QDPR is an enzyme that catalyzes the NADH-mediated reduction of quinonoid dihydrobiop terintetrahydrobiopterin (BH4), a cofactor for phenylalanine hydroxylase, nitric oxide synthase (NOS), and endothelial nitric oxide synthase (eNOS) (27). eNOS is known to catalyze the production of nitric oxide (NO), which diffuses into the vessel wall and leads to relaxation of vascular smooth muscle cells. In addition, it also inhibits the proliferation and migration of smooth muscle cells (28). NO can also regulate vessel function through inhibiting leucocyte adhesion to endothelial cells and migration, as well as platelet aggregation and adhesion (27). Moreover, BH4 deficiency was reported to promote intimal hyperplasia after *in vivo* vascular injury (29). In contrast, sufficient BH4 improved endothelial function in RA patients (30). Of note, QDPR inhibition resulted in a significant increase in TGF-β1 expression in a human kidney cell line (31). Sera of patients with vasculitis, Behcet's disease, and giant cell arteritis showed increased TGF-β1 levels (32, 33, 34). Thus, we hypothesize that QDPR might participate in TAK pathogenesis through BH4/NO or TGF-β1in endothelial cells.

DIXDC1 is an F-actin-binding protein that can regulate the Wnt signaling pathway through inhibiting β-catenin degradation. The Wnt signaling pathway plays an important role in many biological processes, such as embryonic development, cell growth and differentiation, neural development, and cancer cell formation (35). It is also associated with autoimmune diseases, such as RA, SLE, and AS (36).

SPATA7, with an AUC value of 0.803 and 85.4% specificity, was first discovered in primary spermatocytes (37). It is also expressed in the retina, and its mutations are associated with decreased light response (38). SLC25A22 is a glutamate transporter that is located in the inner membrane of mitochondria (39). Mutations in this gene are associated with early infantile epileptic encephalopathy (40). PRH2, which showed 88.3% specificity, is a proline-rich acidic protein, and it can mediate bacteria adhesion to the teeth, resulting in dental caries (41). Human proline-rich proteins are involved in a variety of biological processes, such as regulation of signaling pathways, binding with misfolded proteins, and splicing and processing of RNA (42, 43, 44). ZFAND4, also known as ANUBL1, is a zinc-ion-binding protein that was found to be upregulated in gastric cancer (45). NOLC1 is a highly phosphorylated nuclear protein, which binds to RNA polymerase I when mitosis begins (46). The complex of RNA polymerase I and NOLC1 is able to regulate cell growth and apoptosis (47, 48, 49). Although the functions of these proteins are not thoroughly elucidated, they have the potential to serve as serological biomarkers of TAK due to their high specificity and sensitivity from the arrays.

Compared with each individual marker, the decision tree model showed a higher AUC value at significantly improved specificity. Hence, using the identified panel of biomarkers can potentially facilitate TAK diagnosis. The traditional western blot method for the three representative proteins, SPATA7, QDPR, and PRH2, which had higher AUC among the eight candidates, was proposed for TAK-autoantibody detection and proved the reliability of the candidates. However, it is necessary to validate the eight autoantibodies and autoantibodies identified by decision tree model with ELISA in a larger population to facilitate their applications in a clinical setting. Actually, this is indeed our plan for future studies. In addition, these autoantigens did not differentiate the active and stable groups in TAK diagnosis. Thus, this supports their role as TAK diagnostic markers but not disease activity assessment markers.

We envision that the protein array-based technology has several unique advantages in diagnosis/prognosis in a clinical setting as compared with the traditional methods, such as ELISA- and chemiluminescence-based assays. First, the sensitivity in detecting autoantibodies of the protein array technology is generally higher because of its miniaturized nature. Second, signals obtained on a protein array are quantitative with a wide linear range. Third, it consumes much less samples, which can be critical when the biospecimen, such as spinal fluids and needle biopsy, is difficult to obtain in a large amount. Fourth, the array-based format allows for simultaneously surveying a panel of biomarkers in a highly multiplexed fashion. This is important because we and others have shown that the performance of a biomarker panel is usually significantly better than any of the individual biomarkers in a panel (50, 51, 52, 53). Finally, the protein array-based technology is also amenable to high-throughput platform or even automation.

To our knowledge, this study represents the first proteome-wide, unbiased survey for TAK-associated autoantibodies using a large cohort of 390 subjects. Using a two-phase approach, eight TAK-specific biomarkers were identified, three of which can be readily adopted in clinical practice.

## Data availability

The raw data that support the findings of this study are available from the corresponding author (Peking Union Medical College Hospital, yongzhelipumch@126.com), upon reasonable request.

## Supplemental data

This article contains supplemental data.

## Conflict of interest

The authors declare no competing interests.
