# Supplementary material for: Identification of Novel Serological Autoantibodies in Takayasu Arteritis Patients Using HuProt Arrays
Source: Mol Cell Proteomics. 2021 Feb 3;20:100036. doi: 10.1074/mcp.RA120.002119 (PMC7995655; doi:10.1074/mcp.RA120.002119)
Supplement: Supplemental — Table S1 and Figures S1 to S3 [file mmc1.docx]

**Supplementary table 1. Association between biomarkers and TAK activity in Phase II.**

| Protein | TAK stable (78) | | TAK active (31) | | **χ^2^** | *P* |
| --- | --- | --- | --- | --- | --- | --- |
|  | Positive number | Positive rate (%) | Positive number | Positive rate (%) |  |  |
| SPATA7 | 58 | 74.4 | 22 | 71.0 | 0.13 | 0.72 |
| QDPR | 58 | 74.4 | 20 | 64.5 | 1.06 | 0.30 |
| SLC25A22 | 64 | 82.1 | 24 | 77.4 | 0.31 | 0.58 |
| PRH2 | 33 | 42.3 | 18 | 58.1 | 2.21 | 0.14 |
| DIDXDC1 | 53 | 67.9 | 20 | 64.5 | 0.12 | 0.73 |
| IL17RB | 36 | 46.2 | 14 | 45.2 | 0.009 | 0.96 |
| ZFAND4 | 38 | 48.7 | 17 | 54.8 | 0.33 | 0.56 |
| NOLC1 | 26 | 33.3 | 13 | 41.9 | 0.71 | 0.40 |
| HBEGF | 1 | 1.3 | 2 | 6.5 | - | 0.19 |
| GBE1 | 78 | 100 | 31 | 100 | - | NS |
| SLC27A3 | 1 | 1.3 | 1 | 3.2 | - | 0.49 |
| PLA2G16 | 78 | 100 | 31 | 100 | - | NS |
| PFKFB1 | 6 | 7.7 | 2 | 6.5 | ＜0.0001 | 1 |
| HNRNPC | 1 | 1.3 | 2 | 6.5 | - | 0.19 |
| NME7 | 3 | 3.8 | 2 | 6.5 | 0.001 | 0.94 |
| ESR2 | 3 | 3.8 | 2 | 6.5 | 0.001 | 0.94 |
| UBA1 | 78 | 100 | 31 | 100 | - | NS |
| ACSL4 | 1 | 1.3 | 1 | 3.2 | - | 0.49 |
| CIDEA | 10 | 12.8 | 6 | 19.4 | 0.33 | 0.57 |
| ERMN | 4 | 5.1 | 2 | 6.5 | ＜0.0001 | 1 |
| MAPK1IP1L | 7 | 9 | 2 | 6.5 | 0.002 | 0.96 |
| PAXBP1 | 1 | 1.3 | 1 | 3.2 | - | 0.49 |
| TRIM36 | 1 | 1.3 | 3 | 9.7 | 2.37 | 0.12 |
| NAP1L1 | 44 | 56.4 | 20 | 64.5 | 0.6 | 0.44 |
| PAF1 | 20 | 25.6 | 11 | 35.5 | 1.06 | 0.3 |
| CLPTM1 | 5 | 6.4 | 6 | 19.4 | 2.79 | 0.095 |
| LOXL3 | 20 | 25.6 | 11 | 35.5 | 1.06 | 0.3 |
| SPRR1A | 2 | 2.6 | 1 | 3.2 | - | 1 |
| ARHGAP17 | 2 | 2.6 | 2 | 6.5 | 0.17 | 0.68 |
| SPDYC | 2 | 2.6 | 2 | 6.5 | 0.17 | 0.68 |
| FAM21A | 1 | 1.3 | 1 | 3.2 | - | 0.49 |
| EIF3H | 13 | 16.7 | 7 | 22.6 | 0.52 | 0.47 |
| USF2 | 2 | 2.6 | 2 | 6.5 | 0.17 | 0.68 |
| BC062331.1 | 1 | 1.3 | 1 | 3.2 | - | 0.49 |
| PGPEP1 | 16 | 20.5 | 11 | 35.5 | 2.67 | 0.1 |
| GDE1 | 7 | 9 | 7 | 22.6 | 2.55 | 0.11 |
| CNST | 78 | 100 | 31 | 100 | - | NS |
| HSF2 | 2 | 2.6 | 0 | 0 | - | 1 |
| AGGF1 | 1 | 1.3 | 1 | 3.2 | - | 0.49 |
| SPANXN2 | 16 | 20.5 | 5 | 16.1 | 0.27 | 0.6 |
| NOL3 | 10 | 12.8 | 1 | 3.2 | 1.32 | 0.25 |
| MCM3 | 21 | 26.9 | 8 | 25.8 | 0.01 | 0.905 |
| HSPBAP1 | 31 | 39.7 | 12 | 38.7 | 0.01 | 0.92 |

**Supplementary table 2. Basic characters of subjects in Westerb-blot validation.**

| Subjects (number) | PRH2 | QDPR | SPATA7 |
| --- | --- | --- | --- |
| TAK (3) | 26, 35, 51^a^ | 23, 39, 43 | 19, 33, 49 |
|  | 3/0^b^ | 2/1 | 3/0 |
| HC (3) | 33,40,40 | 35,40,40 | 24,33,40 |
|  | 1/2 | 0/3 | 0/3 |
| RA (2) | 29, 50 | 29/54 | 29/54 |
|  | 1/1 | 2/0 | 2/0 |
| SS (2) | 48, 55 | 48, 55 | 48, 55 |
|  | 2/0 | 2/0 | 2/0 |
| AAV (2) | 51, 54 | 54, 60 | 51, 60 |
|  | 0/2 | 1/1 | 1/1 |

Each table consists of two parts: a, years of old; and b, female/ male. TAK: Takayasu arteritis; HC: healthy control; RA: rheumatoid arthritis; SLE: [systemic](javascript:void(0);) [lupus](javascript:void(0);) [erythematosus](javascript:void(0);); SS: primary Sjögren's syndrome; AAV: ANCA-associated vasculitis.


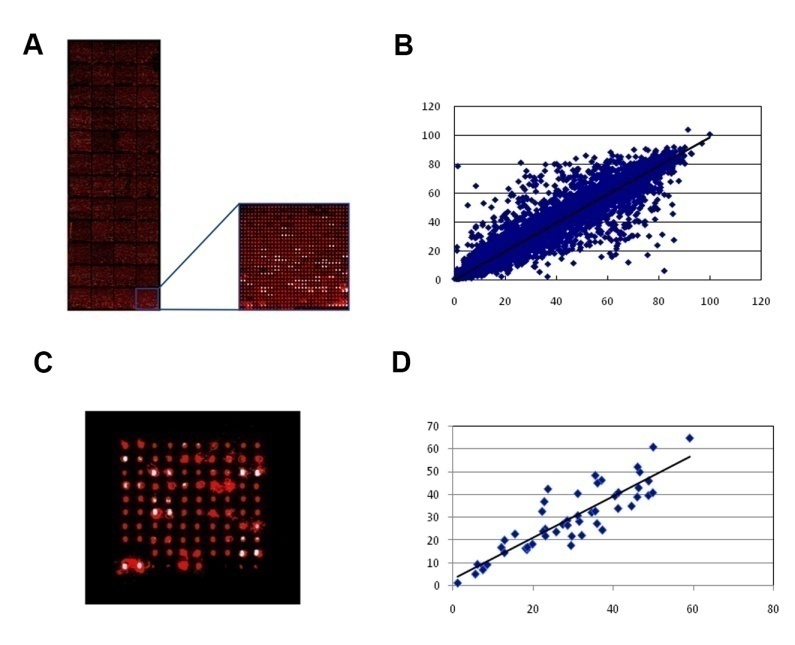


**Supplementary Fig. 1. Quality evaluation of protein arrays.**

(A) Full image of a representative HuProt array probed with anti-GST antibody. On each HuProt array, there are ~20,240 individually purified human proteins located in 48 printing block sand 93.2% of the proteins produced detectable anti-GST signals. (B) The correlation coefficient between duplicate spots for each protein on a HuProt arrayis0.978 (Y = 0.99X + 0.53; R^2^ = 0.96). (C) Full image of a representative sub-array of the 43 candidate biomarker proteins on a TAK-focused array. Each glass slide contains 12 identical sub-arrays. All 43 candidate human proteins were detectable except the negative control. (D) The correlation coefficient between duplicate spots for each protein on a TAK-focused was 0.88, suggesting reliable reproducibility(Y = 0.91X + 2.74; R^2^ = 0.77).


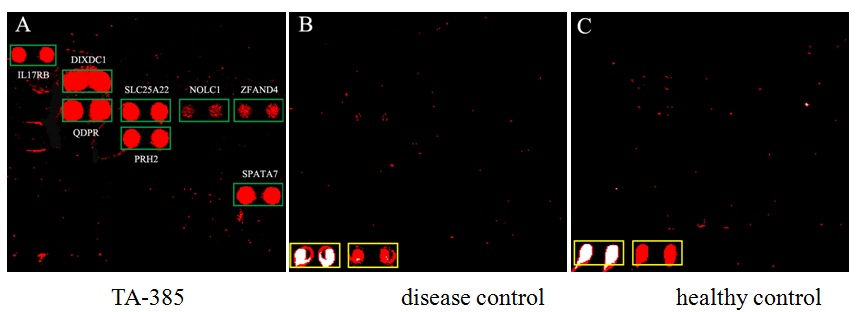


**Supplementary Fig. 2. Eight autoantigens validated in Phase II.**

(A) Representative image of a TAK-focused array probed with a TAK serum sample. Eight autoantibodies can be readily detected (boxed in green). (B-C) None of the eight proteins showed any detectable signals when incubated with a disease control (B) or healthy control (C). Positive proteins that served as landmarks are shown in yellow boxes.


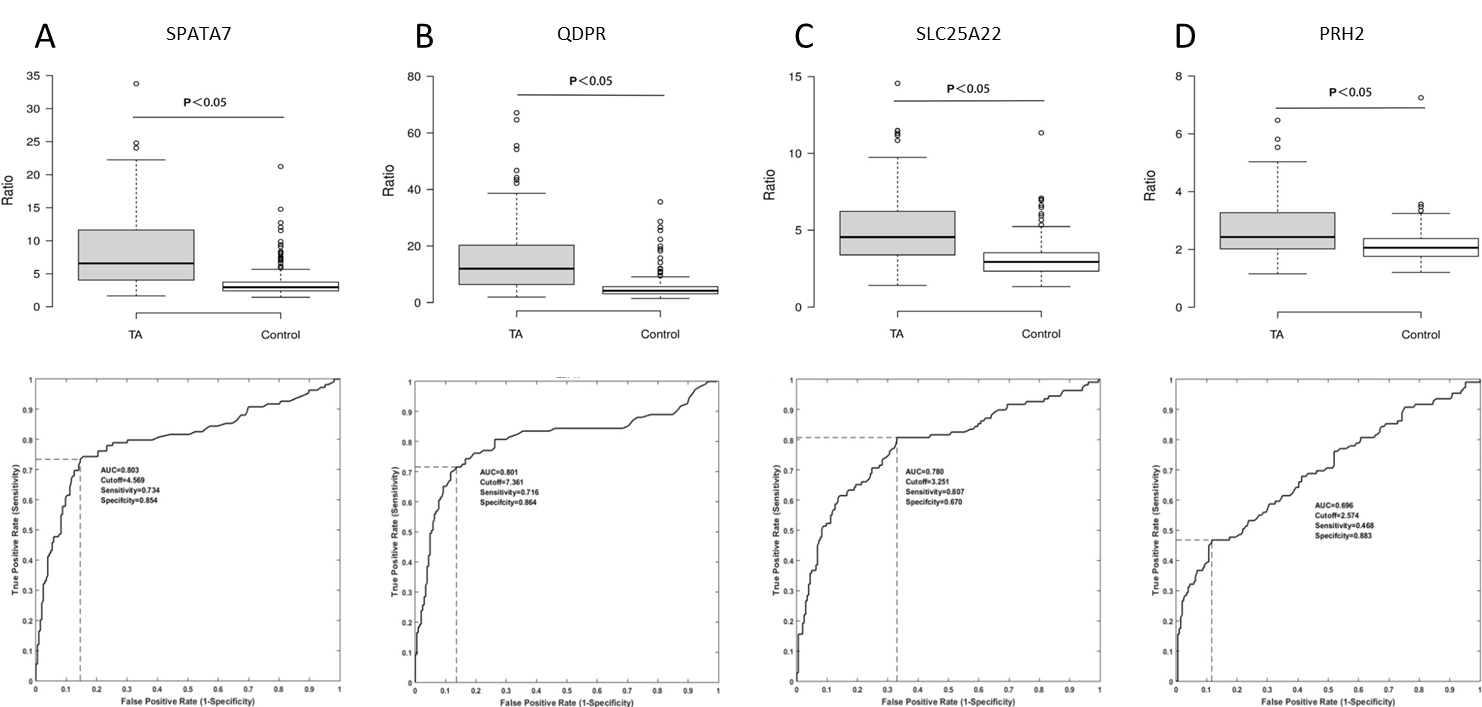


**Supplementary Fig. 3. Boxplot analysis and ROC of the top four autoantigens.**

Boxplot analysis of the top four biomarkers, SPATA7 (A), QDPR (B), SLC25A22 (C), and PRH2 (D), demonstrating that the median values of the signal intensity obtained in the TAK group were significantly higher than that in the negative control group (Upper panel). Corresponding ROC of four autoantigen (Bottom panel).
